# Sliding Speed-Dependent Tribochemical Wear of Oxide-Free Silicon

**DOI:** 10.1186/s11671-017-2176-8

**Published:** 2017-06-12

**Authors:** Lei Chen, Yaqiong Qi, Bingjun Yu, Linmao Qian

**Affiliations:** Tribology Research Institute, State Key Laboratory of Traction Power, Southwest Jiaotong University, Chengdu, 610031 Sichuan Province People’s Republic of China

**Keywords:** Tribochemical wear, Sliding speed, Oxide-free Si, Bond rupture and reformation

## Abstract

Fundamental understanding of tribochemical wear mechanism of oxide-free single crystalline silicon (without native oxide layer) is essential to optimize the process of ultra-precision surface manufacturing. Here, we report sliding speed-dependent nanowear of oxide-free silicon against SiO_2_ microspheres in air and in deionized water. When contact pressure is too low to induce Si yield, tribochemical wear occurs with the existence of water molecules and wear volume decreases logarithmically to constant as sliding speed increased. TEM and Raman observations indicate that the dynamics of rupture and reformation of interfacial bonding bridges result in the variation of tribochemical wear of the oxide-free Si with the increase of sliding speed.

**Electronic supplementary material:**

The online version of this article (doi:10.1186/s11671-017-2176-8) contains supplementary material, which is available to authorized users.

## Background

Material wear can either be a mechanical wear or tribochemical wear depending on the mechanism involved in surface damage [1]. Mechanical wear normally corresponds to fracture, plastic deformation, and viscous flow of materials induced by mechanical impress or/and shear stress [2–4]. By contrast, tribochemical wear is attributed to stress-assisted bond dissociation [5] or along with chemical corrosion in some cases [6]. Single crystal silicon (Si) serves as one of the main material of semiconductor chips [7, 8], and chemical mechanical polishing (CMP) is the most effective approach to manufacture atomically smooth surface for Si semiconductor substrate. The material removal occurring before Si material yield in CMP is generally dominated by tribochemical reaction [9, 10].

CMP is a complicated wear process and susceptible to many factors, such as the material of pad or slurry, and the experimental parameter of load or speed [10]. To simplify the tribological system and identify the wear mechanism in CMP, numerous researches have studied the tribochemical wear of Si against a single SiO_2_ microsphere to simulate the CMP process [11–17]. For example, based on the results obtained in atomic force microscopy (AFM) experiments, a tribochemical wear mechanism is detected that the interfacial bonding bridges formed between individual atoms with the association of water molecules can transfer mechanical energy into Si substrate and then induce Si atoms removal [11, 12]. However, Si samples used in previous tribochemical wear tests normally involve native oxide layer [13–15], which significantly influences Si wear [16]. Few studies have investigated the tribochemical wear of oxide-free Si substrate (without an oxide layer) [17], which is closer to the real CMP process wherein Si surface always keeps fresh state after the oxide layer is removed.

To gain insight into the tribochemical wear mechanism, we investigated the nanowear of oxide-free Si as a function of sliding speed in humid air and in deionized (DI) water. The main finding was that tribochemical wear decreases and then stabilizes as a function of sliding speed in systems with the potential of rupturing and reforming Si_substrate_-O-Si_tip_ bonding bridges between sliding interfaces under the interaction between mechanical stress and water molecules. Fundamental understanding of sliding speed-dependent Si wear mechanism is possibly useful to increase the efficiency of super-smooth surface manufacturing.

## Methods

The samples were p-Si(100) wafers, which surface oxide layer was removed through hydrofluoric acid (40% aqueous solution) etching for 2–3 min following ultrasonic cleaning in methanol, ethanol, and DI water. After removal of surface oxide layer, the root-mean-square (RMS) roughness of Si over a 500 × 500 nm area was 0.12 ± 0.02 nm. Given that the Si surface was terminated by Si-H groups, the sample behaved relatively hydrophobic and its surface showed a static water contact angle of 82° ± 2°. By using AFM (SPI3800N, Seiko, Japan), sliding speed-dependent tribochemical wear of Si rubbed against SiO_2_ microsphere were studied under humid air (RH = 60%) and in DI water. The SiO_2_ with a radius *R* of 1.25 μm was attached to a tip cantilever (Additional file 1: Figure S1 in Supporting Information). The normal spring constant *k* of the cantilever was calibrated to be 10.5–13.8 N/m by using a reference probe (force constant = 2.957 N/m). All nanowear tests were performed at room temperature at an imposed load of 2 μN. The scratch amplitude was 200 nm, and the sliding cycle was 100. The sliding speed ranged from 0.08 to 50 μm/s.

After performing the nanowear tests, the topography of the wear area was imaged by a sharp Si_3_N_4_ tip (*R* = ~10 nm) with a soft cantilever (*k* = ~0.1 N/m) in vacuum (<10^−3^ torr). Wear scars that formed on Si substrate under selected sliding speeds were analyzed by high-resolution transmission electron microscopy (TEM, Tecnai G2, FEI, Holland). Cross-sectional TEM samples were prepared using a focused ion beam system. To maximally reduce the impact of energy-induced decrystallization of Si substrate, we deposited epoxy polymer instead of Pt onto Si surface as the passivation layer during sample preparation. The bonding structure of the original Si surface and wear debris formed in the microwear tests was measured using a Raman spectroscope (RM2000 Renishaw, UK) to detect the possible tribochemical reaction during the sliding process.

## Results and Discussion

### Sliding Speed-Dependent Nanowear of Oxide-Free Si in Aqueous Environments

**Fig. 1:**
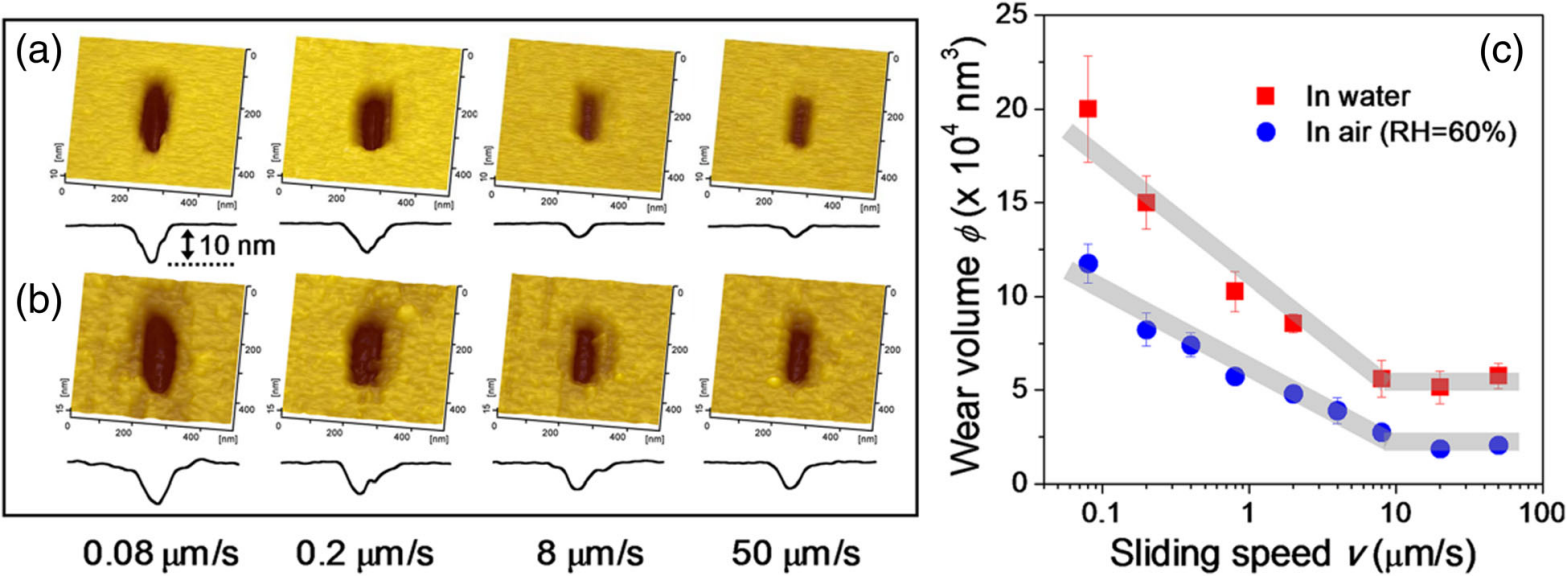
AFM images and the corresponding cross-section profiles of wear scar on silicon surface slid against SiO_2_ tip at sliding speed ranging from 0.08 to 50 μm/s in humid air (RH = 60%) (**a**) and in water (**b**). Volume of wear scars on Si surface as a function of sliding speed in air and in DI water (**c**). Imposed load is 2 μN, sliding amplitude is 200 nm, and the number of sliding cycles is 100

Nanowear of oxide-free Si at various sliding speeds were respectively investigated in humid air (60% RH) and in DI water. Figure 1a, b respectively show the topographic images and corresponding cross-sectional profiles of wear scars. After 100 reciprocating sliding cycles, material removal were observed on oxide-free Si substrates, and a slight wear was detected at high sliding speed (*v*) both under humid air and in DI water. Figure 1c plots the wear volume of oxide-free Si substrate as a function of sliding speed. Under the given conditions, wear volume first decreased logarithmically with increasing sliding speed and then stabilized (~2 × 10^4^ nm^3^ under humid air and ~5 × 10^4^ nm^3^ in water) as sliding speed exceeded the critical value (~8 μm/s).

Under the same load condition, this behavior of sliding speed-dependent oxide-free Si wear was similar to that observed in oxidized Si surface in humid air but not to that observed in DI water [16]. Compared with the oxide-free Si surface terminated with Si-H groups, the surface of oxidized Si is partially covered with silanol (Si-OH) groups, which act as hydrogen acceptor and donor moieties, and the surface displays high potential to absorb water molecules [18]. Study has indicated that too many absorbed water molecules confined between the sliding contact areas may increase the gap between sliding interfaces and prevent removal of Si substrate [16]. In water condition, the surface damage in oxidized Si was completely suppressed. In the present study, after removal of surface oxide layer which occurred in DI water (Fig. 1b), the wear volume was larger in water than that in humid air at each sliding speed (Fig. 1c). In water condition, the energy barrier of tribochemical reaction occurring between Si/SiO_2_ pairs was reduced to be a very limited level [19]. Then, any contact between the SiO_2_ tip and Si substrate with a very small load might cause the material removal of Si surface. This might be the reason that the extra wear traces (outside the wear scar) were observed on Si surface under water condition (Fig. 1b).

### Sliding Speed-Dependent Nanowear of Oxide-Free Si in Dry Air

**Fig. 2:**
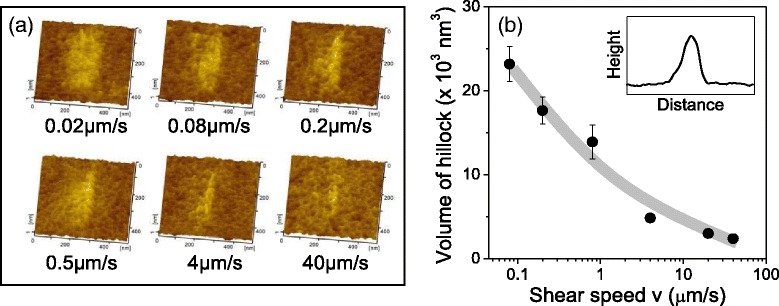
Si wear as a function of sliding speed under dry air. Topography of the wear region (**a**). Volume of hillocks on Si surface formed after 100 sliding cycles in vacuum (**b**). Imposed load is 2 μN, and sliding amplitude is 200 nm. *Inset* in (**b**) shows the diagram of the cross-section profile of a hillock

At an imposed load of 2 μN, the contact pressure estimated by DMT model (<1 GPa) was considerably lower than the yield stress of the Si material (7 GPa) [20]. At this condition, Si wear showed the formation of hillocks instead of material removal at a given sliding speed in dry air (Fig. 2a). Figure 2b (inset) shows the typical cross-sectional profile of a hillock. TEM observations showed that growth of hillocks mainly originated from the mechanical interaction-induced amorphization of Si crystal structure [21]. As sliding speed increased, the calculated volume of hillocks gradually decreased (Fig. 2b), demonstrating the incomplete transformation of Si from crystalline state to amorphous state under high sliding speed [21]. However, this mechanism cannot explain the dependence of Si wear on the sliding speed under humid air or in DI water; Si wear mainly occurred as material removal and not as material deformation. Moreover, these results indicated that material removal under humid air or in DI water (Fig. 1) should be different from the oxidation wear because no groove formed on Si surface though in the presence of oxygen in the atmosphere.

### TEM Observation of Worn Area Formed Under Different Sliding Speeds

**Fig. 3:**
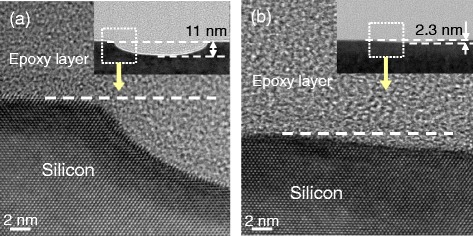
High-resolution TEM images of wear scar on Si substrate formed at sliding speed values of 0.08 (**a**) and 50 μm/s (**b**) in humid air. The *insets* show the wear scars with depths of ~11 nm in (**a**) and 2.3 nm in (**b**)

To reveal the sliding speed dependence of the nanowear mechanism, we characterized the cross-section of wear tracks on Si substrate formed under humid air by using high-resolution TEM. As shown in Fig. 3 (inset), wear scars with depths of ~11 and ~2.3 nm were, respectively, generated under sliding speeds of 0.08 and 50 μm/s. High-resolution TEM images demonstrated that the Si atomic lattice beneath worn surface was organized, i.e., without amorphization or dislocation whether the sliding speed was low (Fig. 3a) or high (Fig. 3b). These results supported the hypothesized tribochemical wear mechanism applied at all sliding speeds, wherein Si_substrate_-O-Si_tip_ bonding bridges formed between sliding interfaces, stripping out the Si atoms from the outermost substrate surface under compression stress and shear stress. Wen et al. [22] recently demonstrated such tribochemical reaction between Si/SiO_2_ sliding interfaces in aqueous environment based on molecular dynamics simulations using ReaxFF reactive force field. During the rubbing process, the contribution of friction heat on the variation in Si wear at different sliding speeds was negligible because the temperature rise was very low at the given conditions [23]. The decrease of Si wear vs. sliding speed (Figs. 1 and 3) also indicated that in humid air (60% RH) or in DI water, the rate of tribochemical reaction changed dynamically with sliding speed.

### Detection of Dehydration and Hydrolysis Reactions Through Raman Analysis

Previous AFM study reported that relative humidity (RH) and sliding speed-dependent tribochemical wear of oxidized silicon were positively correlated with the volume of condensed water bridge when RH is less than 50% [22]. However, this theory cannot be used to explain the variation in tribochemical wear of oxide-free Si substrate vs. sliding speed in water where the number of water molecules in a confined contact area remained constant. Previous studies have detected that the chemical reaction would not take place readily only under mechanical stress and that formation of interfacial bonding bridges is necessary for tribochemical wear to occur on Si substrate [13, 15, 24]. A similar variation in oxide-free Si wear as a function of sliding speed was observed under humid air and in water (Fig. 1), indicating that the tribochemical wear of Si substrate against SiO_2_ tip was directly dependent on the formation of Si_substrate_-O-Si_tip_ bonds with the association of the water molecules. By using kinetic Monte Carlo simulation, Liu et al. [25] verified the occurrence of dehydration reaction between two Si-OH groups on neighbor surfaces, where the Si-O-Si bonding bridge formed and its concentration decreased logarithmically with the increase in sliding speed. As the sliding speed increased, less contact time corresponded to the exponential reduction of Si_substrate_-O-Si_tip_ bonds formed between sliding interface, reducing the tribochemical wear of Si substrate. However, the dehydration reaction should be time dependent. This single theory can fit the logarithmical decrease of wear volume at relatively low sliding speed but cannot explain the constant volume of Si wear at sliding speed values exceeding 8 μm/s.

**Fig. 4:**
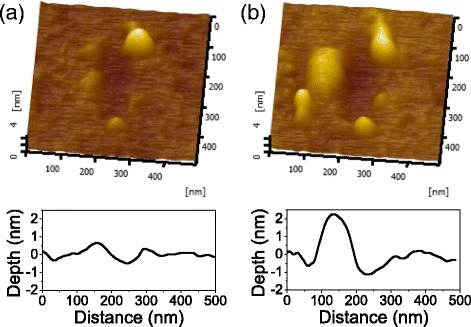
AFM images and the corresponding cross-section profiles of wear scars on amorphous silica surface formed after sliding 200 cycles (**a**) and 2000 cycles (**b**). Relative humidity (RH) was 60%, imposed load was 2 μN, sliding amplitude was 200 nm, and the sliding speed was 0.8 μm/s

Based on the theory of water corrosion, Si-O-Si bonds or Si-Si bonds can be dissociated to form Si-OH groups during hydrolysis reaction [26]. The mechanical pressure or shear stress can deform the Morse potential of bond and lower the energy barrier of bond dissociation, and then bond dissociation is accelerated in tribochemical reaction [27]. ToF-SIMS measurements showed that the peaks of Si-OH and Si-H in wear debris were significantly stronger than those of the original silicon surface [28]. To verify the occurrence of hydrolysis reaction of Si-O-Si bonds during the rubbing process, we investigated the tribochemical wear of amorphous silica slid against SiO_2_ tip at a contact pressure of ~0.7 GPa (total load = 2 μN). As shown in Fig. 4, the grooves with the depth of ~0.5 and ~1.2 nm were, respectively, generated after sliding 200 and 2000 cycles. Since the contact pressure was far less than the yield stress of amorphous silica (8.4 GPa) [20], slight wear of silica formed in humid air verified the occurrence of hydrolysis reaction of Si-O-Si bonds during the rubbing process.

As for the tribochemical mechanism described in this work, it can be reasonably concluded that both dehydration reaction and hydrolysis reaction exist in the chemical reaction between interfacial atoms under a sliding speed. We thus propose that the observed variation in stress-induced chemical wear of Si substrate as a function of sliding speed is the result of rupture and reformation of domains of Si_substrate_-O-Si_tip_ bonding bridges [26–28].

1 $$ {S\mathrm{i}}_{\mathrm{substrate}}{\textstyle \hbox{-}}\mathrm{O}{\textstyle \hbox{-} }{\mathrm{Si}}_{\mathrm{tip}}+{\mathrm{H}}_2\mathrm{O}\overset{\mathrm{Mecahnical}\kern0.5em \mathrm{stress}}{\rightleftharpoons}\kern0.5em {\mathrm{Si}}_{\mathrm{substrate}}\kern0.5em {\textstyle \hbox{-}}\mathrm{O}\mathrm{H}+{\mathrm{Si}}_{\mathrm{tip}}{\textstyle \hbox{-}}\mathrm{O}\mathrm{H} $$

A similar theory that is proposed because rupture and reformation of interfacial H-bond bridges has been successfully used to explain the variation of friction force versus ln[*v*] [29].

**Fig. 5:**
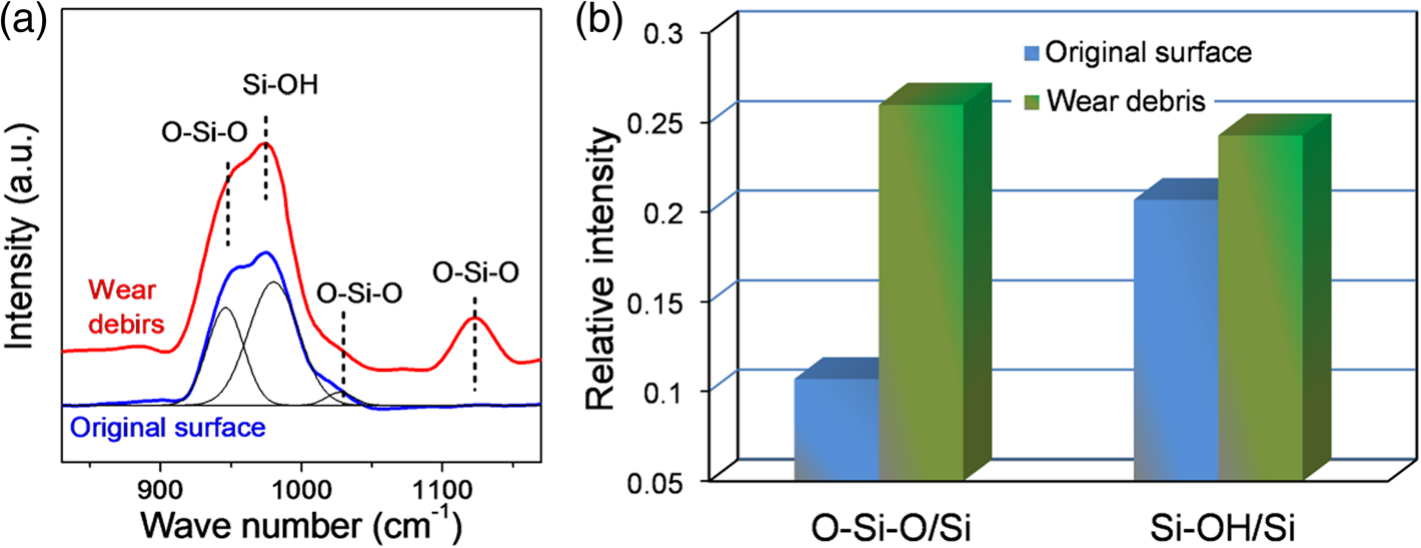
Raman spectra of original Si surface and wear debris at the end of scale formed against SiO_2_ sphere in humid air. **a** Bond detection curves. **b** Relative intensities of O-Si-O/Si and Si-OH/Si estimated from (**a**). Normal load in the wear tests was 1 N, and the number of sliding cycles was 2000

To confirm the occurrence of hydrolysis reaction in tribochemical wear, we prepared a larger scar at microscale on Si surface (Additional file 1: Figure S2b in Supporting Information) against SiO_2_ sphere, and the wear products were analyzed by a Raman spectroscope. In the microscale tests, given that the chosen contact stress was too low to induce mechanical wear of Si substrate under dry air condition (Additional file 1: Figure S2a in Supporting Information), the material removal of Si at microscale should be dominated by tribochemical reaction. It is deduced that the tribochemical reaction occurring during nanowear of Si substrate can be reproduced in the microscale tests. Figure 5a shows the Raman spectra of the original Si substrate and the wear debris on Si surface formed against SiO_2_ sphere under 60% RH air. The characteristic peaks of O-Si-O bonds and Si-OH bond were found in these spectra [30]. Given that the original Si surface and wear debris were exposed to air prior to the Raman measurement, formation of these two bonds on original surface should be attributed to the oxidation and hydrolysis reactions with oxygen and water in air. However, we found that both relative intensities of O-Si-O/Si and Si-OH/Si bonds obviously increased in the wear debris compared with those in the original Si substrate (Fig. 5b). Since the role of oxidation reaction in the formation of wear debris was limited under the given conditions [31], the O-Si-O and Si-OH groups should be generated in the dehydration and hydrolysis reactions.

### Mechanism of Sliding Speed-Dependent Tribochemical Reaction

**Fig. 6:**
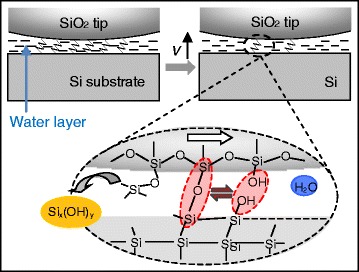
Schematic showing the interfacial state of Si substrate rubbed against SiO_2_ tip under humid air and in DI water with increasing sliding speed *v*

Based on the above discussion, we propose a mechanism for sliding speed-dependent tribochemical wear of Si/SiO_2_ pairs. As shown in Fig. 6, the interfacial bonding bridges of (Si-Si)_substrate_-O-(Si-O-Si)_tip_ is formed under the association of mechanical stress and water molecules. The Si-O bonds (5.82 eV bond enthalpies) of SiO_2_ tip or those in Si_substrate_-O-Si_tip_ bonding bridges are considerably stronger than the Si-Si bonds (2.38 eV) of Si substrate. During the sliding process, both Si-O bonds and Si-Si bonds weakened, but the bonds would rupture preferentially on the side of (Si-Si)_substrate_ with lower energy barrier [32]. No obvious wear of the SiO_2_ tip observed after nanowear tests in humid air and water conditions (Additional file 1: Figure S3 in Supporting Information) also supported this mechanism. At low sliding speed, after stress was released by the slip event, Si_substrate_-O-Si_tip_ bonding bridges reform during dehydration reaction and grow until they become sufficiently large to transmit mechanical stress to Si substrate. When sliding speed is too high or contact time is too low, reorganization will not be completed, resulting in a less stable interface structure and weaker tribochemical reaction. Less amount of reaction products (Si_x_(OH)_y_) formed at high sliding speed [28]. Equation 1 demonstrates a possible explanation to the constant volume of Si wear at relatively high sliding speed (*v* > 8 μm/s), where rupture and reformation of interfacial bonding bridges possibly reach a dynamic equilibrium state (Fig. 6).

Difference in the tribochemical wear in humid air and in DI water (Fig. 1) indicated that reconstitution of Si_substrate_-O-Si_tip_ bonding bridges was closely correlated with environmental conditions. Compared with humid air, DI water contains more water molecules, which are beneficial in hydrolysis reaction as they facilitate the dissociation of Si-Si bonds, resulting in formation of more Si-OH groups on Si surface. A surface containing more Si-OH groups increases the potential of dehydration reaction to form Si_substrate_-O-Si_tip_ bonding bridges that form linkage with SiO_2_ tip surface [32]. As a result, a larger rate of interfacial bonding bridge formation in DI water resulted in more serious tribochemical wear of Si substrate in DI water than in humid air.

## Conclusions

The sliding speed-dependent nanowear of single crystalline Si was investigated in air (0 and 60% RH) and in DI water by using SiO_2_ microspherical tips. The tribochemical wear of oxide-free silicon occurred in the presence of water molecules, and the wear volume logarithmically decreased to a constant with the increase in sliding speed under those two environmental conditions. TEM characterizations confirmed that the subsurface of wear scars were free of mechanical damage under a broad range of sliding speed (from 0.08 to 50 μm/s). Raman analysis indicated that dehydration and hydrolysis reactions both occurred during the tribochemical wear of Si substrate. The dependence of tribochemical wear on sliding speed under humid air and in water can be modeled using stress/water-associated interfacial bond formation kinetics; interfacial reaction occurs via formation and rupture of Si_substrate_-O-Si_tip_ bond bridges between Si substrate and SiO_2_ tip contact surfaces, resulting in the variation in tribochemical wear on Si surface as a function of sliding speed. This study provides further insight into the tribochemical wear mechanism of Si CMP, which is of great significance for improving polishing efficiency. For example, restraining the hydrolysis reaction of Si-O can advance the tribochemical removal of Si materials, which may help to explain why the optimal pH of alkaline slurry is 10–10.5 in the CMP process.

## Additional file

Additional file 1: Supporting information. **Figure S1.** SEM and AFM images of SiO_2_ microspherical tip. **Figure S2.** Three-dimensional morphologies and their corresponding cross-section profiles of Si wear formed in dry air (a) and in humid air (b). **Figure S3.** AFM images and profiles of SiO_2_ microspheres used in nanowear tests. (a) before nanowear tests; (b) schematic of tip-scanning-tip method; (c) after 100 sliding cycles in humid air; (d) after 5000 sliding cycles in humid air; and (e) after 5000 sliding cycles in DI water. Profiles of SiO_2_ microsphere before and after nanowear tests are indicated by thin black lines and thick red lines, respectively. (PDF 1.24 mb)

## Abbreviations

AFM: Atomic force microscope
CMP: Chemical mechanical polishing
DI water: Deionized water
RMS: Root-mean-square
TEM: Transmission electron microscopy
ToF-SIMS: Time-of-flight secondary ion mass spectrometry
