# Supplementary material for: Sliding Speed-Dependent Tribochemical Wear of Oxide-Free Silicon
Source: Nanoscale Res Lett. 2017 Jun 12;12:404. doi: 10.1186/s11671-017-2176-8 (PMC5468177; doi:10.1186/s11671-017-2176-8)
Supplement: Additional file 1: — Supporting information. Figure S1. SEM and AFM images of SiO2 microspherical tip. Figure S2. Three-dimensional morphologies and their corresponding cross-section profiles of Si wear formed in dry air (a) and in humid air (b). Figure S3. AFM images and profiles of SiO2 microspheres used in nanowear tests. (a) before nanowear tests; (b) schematic of tip-scanning-tip method; (c) after 100 sliding cycles in humid air; (d) after 5000 sliding cycles in humid air; and (e) after 5000 sliding cycles in DI water. Profiles of SiO2 microsphere before and after nanowear tests are indicated by thin black lines and thick red lines, respectively. (PDF 1.24 mb) [file 11671_2017_2176_MOESM1_ESM.pdf]

Supplementary Material for

Sliding speed dependent tribochemical wear of silicon without native oxide layer

Lei Chen, Yaqiong Qi, Bingjun Yu, Linmao Qian*

Tribology Research Institute, State Key Laboratory of Traction Power, The School of Mechanical Engineering, Southwest Jiaotong University, Chengdu 610031, China

E-mail: linmao@swjtu.edu.cn

I. Characterization of SiO_2 micro-tip used in the nanowear test

Before nanowear tests, the SiO_2 microspherical tip was characterized by SEM and AFM. Figure S1 shows the SEM images of SiO_2 tip (left picture) and silica micro-ball (middle picture) attached at the end of silicon cantilever, as well as the topography on its top surface over 200 nm^2 area where the root-mean-squares (RMS) roughness was identified as around 0.4 nm.

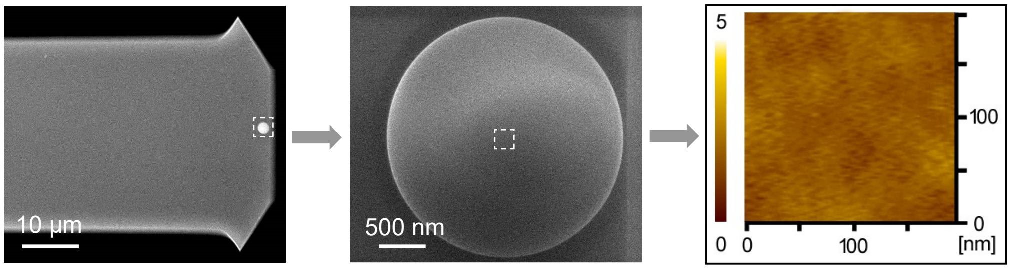

Figure S1. SEM and AFM images of SiO_2 microsoherical tip

II. Microwear of Si substrate in nitrogen and in humid air

The micro-wear of Si substrate against SiO_2 sphere (diameter = ~3 mm) pairs were performed by a probe-type wear test apparatus which had been reported previously. The tests were separately conducted in dry air and in humid air (~50% RH). The applied load was 1 N and the number of sliding cycles was 2000. The reciprocating displacement amplitude D was 100 μm and the sliding speed was 1.2mm/s. Figure S2 shows the topographies and their corresponding cross-section profiles of Si wear formed in dry air and in humid air. Since the contact pressures (< 1 GPa) at the load of 1 N was far less than the mechanical stress corresponding to the yield of Si material (~11 GPa) [1], the mechanical interaction could not induce the wear of Si substrate and without surface damage was observed in dry air (Figure S2a). When the wear test was operated in humid air, material removal occurred on Si surface against SiO_2 sphere (Figure S2b). The results indicated

that the wear of Si substrate generated in the microwear test under humid condition should be dominated by tribochemical reaction [2].

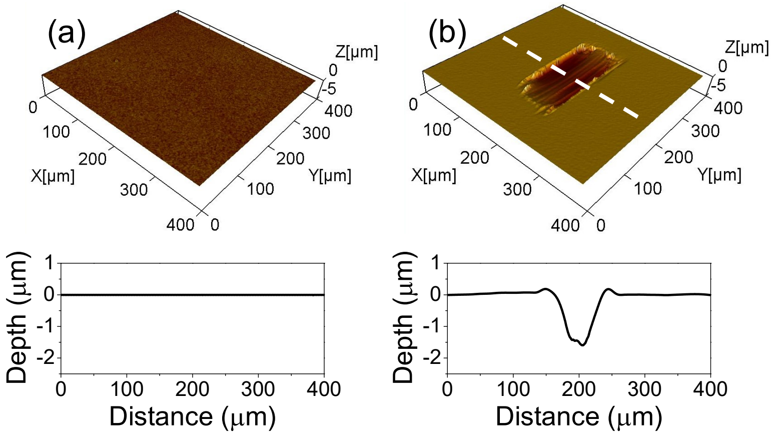

Figure S2. Three-dimensional morphologies and their corresponding cross-section profiles of Si wear formed in dry air (a) and in humid air (b).

II. Characterization of tip wear in tribochemical reaction

The topographies of the SiO_2 tips were compared before and after the nanowear tests. Figure S3a shows the AFM images of a new SiO_2 tip obtained through using a Si_3N_4 tip (R = ~10 nm) scanning on the surface of SiO_2 microsphere (Figure S3b), rather than the tested SiO_2 tip directly scanning a grating sample with an array of sharp tips. The Si_3N_4 tip with softer cantilever can control the minimum contact load within ~2 nN (~150 nN for SiO_2 tip) during scanning process, thereby maintaining all topographical details on the SiO_2 tip surface, such as the adhesive wear debris generated from Si substrate.

Figure S3c shows the AFM image and the corresponding cross-sectional profiles of SiO_2 tip after 100 reciprocating sliding cycles under humid air. The perfect profile indicated that no obvious wear formed on SiO_2 tip surface, and the observed wear debris should mainly come from Si substrate in tribochemical wear. When the number of sliding cycles increased to 5000, a platform formed in the contact area on SiO_2 tip surface as shown in the AFM images (Figure S3d). However, coincidence

between the profiles obtained before and after the tests was mainly attributed to the adhesion of wear debris from Si substrate and not on wear of SiO_2 tip. Similarly, SiO_2 tip maintained its perfect structure after 5000 sliding cycles in DI water, and the tip was covered with low amount of wear debris (Figure S3e).

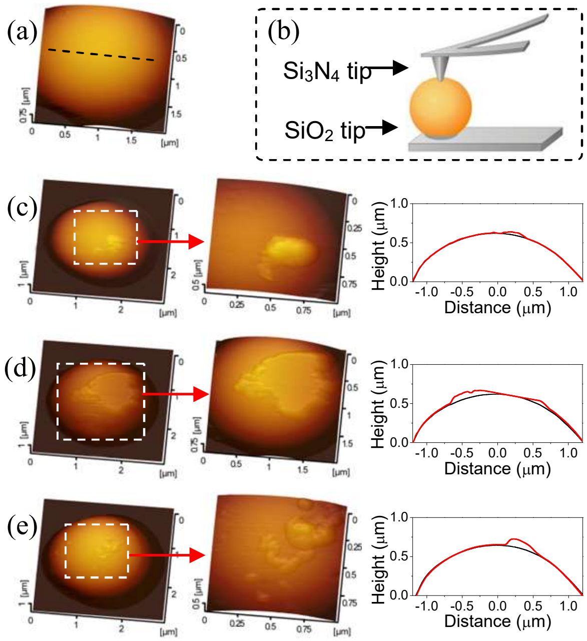

Figure S3 AFM images and profiles of SiO_2 microspheres used in nanowear tests. (a) before nanowear tests; (b) schematic of tip-scanning-tip method; (c) after 100 sliding cycles in humid air; (d) after 5000 sliding cycles in humid air; and (e) after 5000 sliding cycles in DI water. Profiles of SiO_2 microsphere before and after nanowear tests are indicated by thin black lines and thick red lines, respectively.

References

1. Schwarz DA, A generalized analytical model for the elastic deformation of an adhesive contact between a sphere and a flat surface. J Colloid Interface Sci 2003, 26:99-106.
2. Yu JX, Kim SH, Yu BJ, Qian LM, Zhou ZR, Role of tribochemistry in nanowear of

single-crystalline silicon. ACS Appl Mater Interfaces 2012, 4:1585-1593.
